# Manganese in Drinking Water: Higher Doses May Hamper Intellectual Function

**Published:** 2006-01

**Authors:** Dinesh C. Sharma

Manganese is an essential nutrient for humans, but its excessive consumption can cause adverse health impacts. Past studies have linked inhalation of excessive manganese to neurotoxicity in adults. Now a group of U.S. researchers suggests that ingesting high doses of manganese in drinking water can hamper intellectual function in children **[ *EHP* 114:124–129]**. These effects were seen most strongly on scales that measure performance aspects of intellectual function.

The same group had earlier observed a negative impact of water arsenic on intellectual function among children in Araihazar, Bangladesh. Though the manganese concentration in the water these children drank was much higher than its arsenic content, the independent impact of manganese on intellectual function could not be verified. The present study included 142 10-year-old children (including 54 children from the earlier study) who consumed well water with average concentrations of 793 micrograms per liter (μg/L) manganese and 3 μg/L arsenic.

The children’s intellectual function was assessed on six tests (similarities, digit span, picture completion, coding, block design, and mazes) drawn from the Wechsler Intelligence Scale for Children, Version III. Results were summed to create Verbal, Performance, and Full-Scale raw scores. These tests were chosen as they could be applied to Bangladesh’s rural context with minimal alteration. The results showed that manganese concentration had a significant negative dose–response association with all three raw scores.

The researchers found that children in exposure groups 1 (manganese lower than 200 μg/L) and 4 (manganese higher than 1,000 μg/L) differed significantly from one another for Verbal, Performance, and Full-Scale raw scores. Compared to group 1, children in exposure groups 2 (manganese between 200 μg/L and 500 μg/L) and 3 (manganese between 500 μg/L and 1,000 μg/L) had lower Full-Scale and Performance scores, but the differences were not statistically significant. Verbal scores of the children in groups 2 and 3 also did not differ significantly from those in group 1. Due to the lack of measures of intelligence standardized for use in Bangladesh, the team could not calculate IQ points lost.

Though the children’s waterborne manganese intake was lower than the highest safe daily dose (6 milligrams per day) estimated by the U.S. Institute of Medicine, the authors write that additional dietary exposure could have pushed the total daily dose above this value. Moreover, bioavailability of manganese from food is very low, while it is high from drinking water. This could have contributed to neurotoxicity seen in children drinking water with higher amounts of manganese.

The authors point out that their findings are relevant in the United States as well. Data collected by the U.S. Geological Survey have shown that about 6% of domestic wells contain manganese concentrations higher than 300 μg/L. Based on these data and their study results in Bangladesh, the researchers suggest that some U.S. children may be at risk for manganese-induced neurotoxicity.

## Figures and Tables

**Figure:**
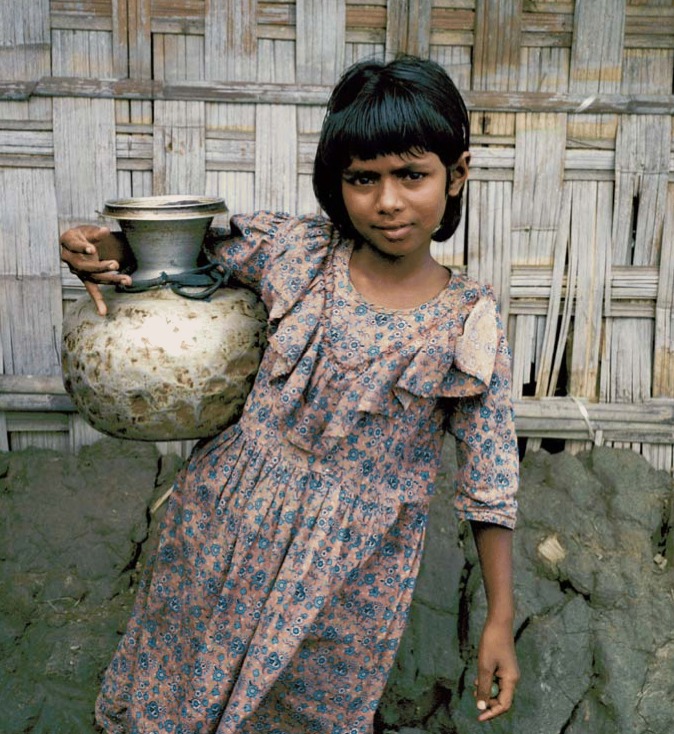
Toxics, toxics everywhere . . . Many studies have looked at the health effects of arsenic in Bangladeshi well water. New data now show that manganese in the water may also cause adverse effects.

